# Efficacy of Single Administration of High-dose Phenobarbital Suppository as Initial Therapy for Benign Infantile Convulsions with Mild Gastroenteritis

**Published:** 2018-04

**Authors:** George IMATAKA, Shigemi YOSHIHARA

**Affiliations:** Dept. of Pediatrics, Dokkyo Medical University, Tochigi, Japan

## Dear Editor-in-Chief

Benign convulsions with mild gastroenteritis (CwG) are situation-related seizures characterized by a cluster of seizures, which are sometimes induced by pain, and/or crying. The clinical features of CwG include (a) afebrile generalized seizures associated with gastroenteritis in previously healthy patients between six months and three years of age; (b) seizures frequently occurring in clusters; (c) normal laboratory tests including serum electrolytes, glucose, and cerebrospinal fluid; (d) normal interictal electroencephalogram (EEG); and (e) good outcomes following seizures and excellent developmental outcome (1–3). This pathological condition occurs in 2.6% of Japanese patients with rotavirus infection (4). On the other hand, there are few reports of CwG from other than Asians (4–6), the cause of which is uncertain. We hypothesized that sodium channels are involved in the onset mechanism of benign infantile convulsions with mild gastroenteritis (CwG). Therefore, we devised a study using initial therapy for high dose phenobarbital (PB) with sodium channel inhibitory effect on CwG. We studied the results of two treatment protocols for CwG that we enforced.

To prospectively investigate the effect of high dose PB treatment on CwG, we first performed PB suppository for the first time and then PB oral therapy for 3 days (Protocol 1: 10 mg/kg PB suppository was initially administered, and 5 mg/kg was added 30 min later. Two doses of 5 mg/kg/day of PB powder were administered orally on Day 2 to day 4 from the onset). As a result of protocol 1 treatment for CwG exhibited good effect. Thus, for that reason, we performed further simplified treatment protocol using single time PB high dose (Protocol 2: A 10 mg/kg single dose PB suppository therapy was administered and observation). Protocol 1 compared 15 cases of CwG treated at Dokkyo Medical University Hospital during an epidemic season for both norovirus and rotavirus infection between October 2006 and September 2007. A high-dose (10 mg/kg) PB suppository was initially administered followed by a 5 mg/kg suppository 30 min later. Patients then received 2 doses of 5 mg/kg/day of PB powder orally from day 2 to day 4. The blood PB concentration was tested 24 h after the administration of the second, 5 mg/kg PB suppository. All patients underwent brain CT on the first day of CwG. EEG was performed both on the first day of therapy and 14 days after the onset of CwG to evaluate the effects of the treatment on convulsions. This protocol was carried out in in-patients department of our hospital. All patients gave informed consent and the study was approved by the hospital.

In protocol 2, a high-dose (10 mg/kg) PB suppository was administered once to patients with CwG, treated at Dokkyo Medical University Hospital during an epidemic season for both norovirus and rotavirus infection from October 2007 to September 2009. The blood levels of PB were examined 24 h after the treatment. This protocol was carried out in outpatients’ department of our hospital (Table 1).

**Table 1:** Therapy with high-dose phenobarbital (PB) suppository in both study (protocols 1 and 2)

***Protocol 1, steps***	***Protocol 2, steps***
Initial treatment:	Treatment:
High-dose PB suppository (10 mg/kg)	High-dose PB suppository (10 mg/kg)
Follow-up treatment, 30 min later:	No follow-up treatment
Intermediate-dose PB suppository (5 mg/kg)	
Follow-up treatment, days 2 – 4 after initial therapy:	
Oral administration of PB powder (5 mg/kg b.i.d.)	

The diagnosis of CwG was based on the criteria (2). CwG was diagnosed when a patient showed seizures associated with gastroenteritis without clinical signs of dehydration or electrolyte derangement, and with body temperature of < 38.0° C before and after seizures. Protocol 1 was effective for CwG in all 15 patients. The average of blood PB concentration was 15.1μg/ml 24 h after the initiation of therapy. Eight patients were able to sleep for at least 4 h after receiving the first two suppositories. Protocol 2 was administered and CwG was controlled in all 10 patients. The average blood PB concentration was 12.1 μg/ml at 24 h after the initial treatment.

This study demonstrated the efficacy of high single-dose PB suppository treatment as an initial therapy for CwG. We conclude this PB therapy is therefore strongly recommended for the treatment of CwG.
